# An Ishmaelite

**Published:** 1888-08-11

**Authors:** Leslie Keith

**Affiliations:** Author of "The Chilcotes," "Uncle Bob's Niece," "East and West," etc.


### An Ishmaelite.

By Leslie Keith,

Author of "The Chilcotes," "Uncle Bob's Niece," "East and West," etc.

(*Continued from p.* 298.)

Chapter XVIII.—The Pariah and his Kindred.

The house in Pont Street was open again in October; the brown-holland pinafores were removed from the furniture, and the sun looked in at the windows and saw Nellie and her baby and her husband – the two first very often together, the two last less often,

There were few balls or dinners at this season, but Richard Bellynge was a man who liked society, and who found it at all times of the year.

The more fortunate of the relations who had country places remained in them till Christmas should be over, and Nellie was thus delivered from the necessity of making a perpetual round of calls.

The little victoria still came daily to the door and took Nellie out; but she went languidly, and because it was less trouble to go than to invent an excuse for staying at home.

A shadow had somehow fallen between Nellie and her husband, and it kept them apart. That frightened act of disobedience Nellie had allowed herself when she wrote to Fred, had never been discovered; but the fear that it might yet be found out lay heavily on her timid spirit. The consciousness of even a little secret such as this was enough to make Nellie constrained and uneasy; and Richard Bellynge, having no clue to this mood, was ungentle and impatient over it.

"What's the matter with you?" he would ask. "Why do you look like that?"

And when Nellie said that there was nothing the matter, he looked as if he doubted her. She grew to shrink from the inquisition of his glances, and to escape from his company when she could. And in mystified disgust and dudgeon he often enough left her when he might have been with her, and dined at his club rather than at home.

Fred had sent no reply to Nellie's impulsive little letter. If he had answered it at once it might have been better for her; for she brooded over his silence, and every day found a new reason for it. The shock of hearing that Fred was living in London—a free man once more—had sent Nellie's thoughts forcibly back on the past days of youth, where her brief romance had been played out. In her present somewhat strained relations with her husband, it was natural enough that she should look back on that short episode as the brighest and most beautiful of her life, and think that in losing Fred she had lost all her best chances of happiness.

It was quite true, as she told her husband, that he could not look on Fred as she and Arthur looked on him; to Richard Bellynge Fred was simply a baddish young fellow, with vicious tendencies, which his weakness of character had developed and confirmed—a scamp, in short; but to Nellie and to Arthur he was the dear companion of their childhood, the bright and loving boy, the handsome, clever, winning young fellow, whose very faults were but another appeal to their affection. Both were right, no doubt, since each saw but one side of a complex character.

But Nellie was treading on a dangerous margin when she began to cast longing eyes on the past, and to wonder whether it would not have been better to have been less faint-hearted and to have waited—or, at least, not to have married.

All unconsciously Richard Bellynge helped to widen the breach.

"Nellie," he said, one morning; he spoke a little curtly, for her nameless depression annoyed him. "I want you to go and dine with me in Grosvenor Place to-night. Christina and Tom are in town for a night on their way to Cannes."

"Oh, I don't want to go," said Nellie, her strong dislike giving her courage; "and—I thought you were going to your club."

"Why don't you want to go?" demanded Richard, looking up from his newspaper with a frown.

"I—I am tired," said Nellie, "and I don't get on with Christina; she always frightens me."

This line of argument was, of course, incomprehensible to a man. Christina did not frighten *him*. He thought her a fine, dashing, agreeable woman, who had got into a capital set and had the wit to keep in it, and he could not guess how such society might be too cruelly genteel for his timid little wife.

"If you have no better reason than that," he began rather sternly, and then Nellie faltered out:—

"Arthur was coming to dine with me; I have not seen him since we came home."

"Arthur? Nonsense," said Bellynge, impatiently. "Your brother can dine with you any day. He seems to have time enough for all his philanthropic fads, and if that's what you talk about when he comes, I should say it would keep. You'll oblige me by fixing another day for your brother. I promised Christina I would bring you."

Nellie submitted. It was always very easy to cow this little woman into obedience; but even a worm may have its private resentments.

"If you will write a line I will post it as I go out," said Richard, willing to be gracious now that he had conquered. "It will save him coming here for nothing."

Nellie went over to her writing table and penned a few lines to her brother with a hand that trembled in sympathy with her throbbing heart. She was as angry as it is given to a meek creature to be; but she put none of her foolish disappointment in her note.

"Dear Arthur," she wrote, "I find Richard has made an engagement for me this evening. Will you dine with us another day instead?"

She handed the sheet silently to her husband, and he glanced at it before putting it into his pocket.

"All right," he said. "I'll send it by messenger, and then he'll be sure to get it in time."

Nellie said nothing, but she did not go to the door to see her husband off, as she had formerly done. Those trifling acts of wifely devotion that tell so accurately the condition of the family barometer were now one by one omitted by her; her husband went off in the morning without a farewell, and his return in the evening was scarcely noticed by her. The barometer at Pont Street stood at Change; but a trifle more and it would reach the region of disastrous storms.

When Richard went off Nellie drew a chair to the fire and sat there nursing her spark of anger till it grew into quite a respectable flame.

Why should Richard's brother be more important than hers; why should Arthur be sacrificed to Tom? "We are always having Tom, or Henry, or Jim, and I am expected to give up everything for them, and when my own brother comes—and he comes so seldom—Richard is quite rude to him."

The jealous tears rose to Nellie's eyes, and that dangerous vision of the past, when she had had Arthur and Fred and her aunt to love and make much of her, rose once more upon her mental horizon. The tears fell when she remembered how she had counted on that evening, when Richard should be at his club and she should have Arthur all to herself, she had meant to ask him so many things about—about Fred.

When Nellie reached this crisis in her emotional tumult, a temptation suddenly dangled itself before her. Our temptations know our weak moments unerringly. Why should she not go to see Fred? Was she to be cut off from her own people altogether? Was Richard to have his own way in everything.

The veriest trifle sometimes gave the deciding impulse that sways our action this way or that. The footman coming in to put coals on the fire caused his young mistress to look up. Seeing the man she gave utterance to the wish she had just formed, and so, as it were, put it beyond risk of change.

"Tell Briggs I shall want the carriage to-day at twelve."

The dinner hour in Gillespie Street was one o'clock. This alone, in the eyes of certain people, would have accurately defined the social position of the Proudies and their neighbours. The people who dine at one and have suppers or meat teas are not the people whom you expect to meet in the genteel world; Nellie did not know to what she was going when she descended from her carriage a street's length off, and timidly asked her way to the Proudie establishment. But she thought, perhaps, that the people would be rude and vulgar, and would stare and make free remarks and drop their h's and add on superfluous r's, "and oh, what misery that must be to one so refined as Fred!"

The ways and the look of the streets were all unfamiliar to her, quite as unfamiliar as was her own bit of London to many whom she met; her heart beat fast as she went. How would Fred receive her? Would he listen if Nellie begged him to come away from this dreadfully dreary place?

What Nellie meant to do with him, if she had persuaded him to go back into her own region, she did not know or consider; she only knew that she was going to see Fred—her dear old playmate—her lover in happier days, and that Richard had been very unkind.

The eating-house was sending forth a pungent, steamy odour of soup and stews; customers were jostling each other briskly, studying the bill of fare (written in Delia's round-hand) in the window, or trooping within as this delicate, frightened little lady went by. She faltered before it an instant, seeing the name she sought written above the door, but her courage was not robust enough to carry her over the threshold. Then she noticed the same name inscribed on the neighbouring shop, and she remembered that Arthur had spoken of a stationer's; yes, there were books and a packet of paper in the window.

Gathering up all her boldness, Nellie mounted the steep, worn steps, and pushing open the glass door with the warning-bell, found herself face to face with Fred.

They stood for a moment looking at each other in a stupefied wonder. Then slowly recognition and something like terror grew in Nellie's eyes; this grey and haggard and worn-looking man—could it be the Fred of her dear remembrances?

"Fred," she said, almost in a whisper, "don't you know me? I am Nellie." Her hands went fluttering out to him.

They fell back piteously, however, before the look on his face. A whole history was written in the changes of his expression. When he lifted his eyes and saw Nellie, so little changed with the passage of the years, it was as if he saw his own past life—his own lost chances rising up before him. Nellie belonged to the time before—before he had fallen, she had nothing to do with him now.

The lines about his mouth grew stern as he remembered this; and it was this stern look before which Nellie shrank abashed.

"Fred," she said imploringly, "have you forgotten me? I have never forgotten you—and it has seemed such a long, weary time——"

"You have no right to be here," said Fred, at last. "This is no place for you. Or—have you come to see the library? The—the books that Arthur chose?"

He looked round him desperately and saw Barbara at her desk—a wondering spectator of the little scene. In another moment Barbara would have slipped away, as she often did when Arthur came; but Nellie's entrance had been so sudden that she had had no time to collect her thoughts.

"You have come to see the books," said Fred. "Miss Proudie will show them to you." He turned a white, beseeching face to Barbara.

"Oh, Fred, it was you I came to see," said Nellie. The sweet mouth drooped and the tears trembled on the dark lashes. "It is such a long time, and you did not answer my letter."

She took no notice of Barbara, who had left her desk, but was standing far off, so that they might talk if they would without her overhearing their words.

"No," he said, commanding himself with a great effort and speaking coldly; "I did not answer your letter, Mrs. Bellynge."

"Mrs. Bellynge!" said Nellie, with dismay and reproach in her voice. "Oh, Fred, do you want me to forget my own people too, as my husband does? Do you think because—because I married that I have no heart or memory? My husband cannot keep me from remembering. He may be angry when he hears that I came, but he can't take away all my past from me. We were friends long ago, and though you went away I have not forgotten."

Fred went a step nearer.

"I think he would like best if you were to go home," he said. "There is nothing to see here after all, except some volumes. This is the dinner hour; but in a little while the readers will be coming in for their books and papers, and you may find them rough and uncivil. You have left your carriage somewhere near?"

"Fred," said Nellie, falling back a step, and wearing a bewildered look that was very pathetic, "are you going to send me away without a word, as if I were a stranger; when I have dreamed so long of telling you that—that I have been so unhappy? You have changed outwardly. Have you changed inwardly too, and forgotten?"

"Yes," said Fred, "I have forgotten. The Fred you speak of died a long time ago. Think of him as dead. I am in charge of the library here; I help this lady. She will go with you, I think, to where your carriage is, as the streets are strange to you, and the workpeople will be coming out presently."

He looked at Barbara with an appeal in his face to which she did not refuse to respond. She came forward silently.

"Let me show you the way," she said. There was perhaps a touch of haughtiness in her voice.

Nellie did not hear the offer. She looked at Fred—a curious, wistful, half-scared look, which he rembered long afterwards, and then, without a word, she tottered down the steep steps to the street.

Barbara was at her side in a moment.

"Will you take my arm?" she asked; but Nellie refused it petulantly.

"I can go alone," she said. "I came alone, and—and I had better have stayed away."

"Mr. Eastgate will expect me to see you safely to your carriage," said Barbara coldly. "Do you remember where you left it?"

Nellie pointed to a turning a little way off, and thither they directed their steps, both silent; pride in both hearts, wounded love and anger in the one.

Thus the dignified coachman and footman, who had expended some wonder over the freak that brought their young mistress to this ungenteel region, saw her coming back to them, walking side by side with a tall young person who looked like a lady, though she wore neither hat nor gloves, and consequently could not have been one.

Nellie stepped into the little Victoria, and said "Home" in a sharp voice to the footman, and was whirled away without any parting word or goodbye for her guide.

It was a very unhappy lady, pretty and graceful though she was, and young too, that the quick-stepping horses bore away from Bermondsey. As for Barbara, her smooth head was held very erect as she returned to her task. She did not go back to the library; there was a private door that led to the rooms above, and finding the key in her pocket she entered the house that way and went to her room.

*(To be continued.)*

